# Volatilome in Milk for Grana Padano and Parmigiano Reggiano Cheeses: A First Survey

**DOI:** 10.3390/vetsci6020041

**Published:** 2019-05-09

**Authors:** Massimo Faustini, Giovanni Quintavalle Pastorino, Carla Colombani, Luca Maria Chiesa, Sara Panseri, Daniele Vigo, Giulio Curone

**Affiliations:** 1 Dipartimento di Medicina Veterinaria, Università degli Studi di Milano, 20133 Milano, Italy; g_pastorino@hotmail.com (G.Q.P.); carla.colombani@unimi.it (C.C.); daniele.vigo@unimi.it (D.V.); giulio.curone@unimi.it (G.C.); 2 Dipartimento di Scienze Veterinarie per la Salute, la Produzione Animale e la Sicurezza Alimentare, Università degli Studi di Milano, 20133 Milano, Italy; luca.chiesa@unimi.it (L.M.C.); sara.panseri@unimi.it (S.P.)

**Keywords:** bovine, milk, Parmigiano Reggiano, Grana Padano, volatiles

## Abstract

Milk characteristics in terms of volatile compounds can influence the subsequent product characteristics and can give indications about metabolism. These features can strongly depend on feeding and management. In this perspective, the screening of milk samples intended for Parmigiano-Reggiano and Grana Padano located in Northern Italy was performed, focusing on a panel of volatile molecules. The work was carried out on a total number of 25 bovine milk samples for the production of Parmigiano-Reggiano and Grana Padano. Milk samples were collected from May to September and analyzed for volatile molecules using headspace gas chromatography mass spectrometry. A range of several volatile molecule classes was considered (aldehydes, ketones, alcohols, carboxylic acids, esters, aromatic hydrocarbons, solforates). Results showed a significant influence of the month and destination of milk due to the time period and subsequent use in cheesemaking (Parmigiano-Reggiano and Grana Padano). Significant differences between months were observed for all volatiles. These preliminary results indicate differences between the two types of milk due to the period and destination. The study of volatile molecules in milk will give important information about the physiology of milk and the evolution of dairy products. These features must be extended and confirmed by the sensory analysis of milk and derived products, leading to a more complete characterization of milk biology and derived products.

## 1. Introduction

Ruminant milk has different physical and chemical characteristics involving differences in sensory properties [1]. Flavor is probably the most relevant attribute in milk preference, and the milk flavor is the result of a balance of volatile compounds present in a broad concentration spectrum in raw milk.

There is a complex interaction between animal metabolism, the environment, and the involved molecules implied in the definition of flavor and aroma [2]; milk aroma is characteristic for each specie, and is strictly related to the volatilome footprint [3]. The environmental changes and the treatment imposed to milk production can deeply modify the volatile compounds’ profile with the possible amelioration of flavor or the appearance of off-flavors.

While it is well-known that small ruminant milk differs from cow milk, discrepancies can be appreciated also between bovine breeds. Flavor compounds derive from fodder (direct passage), rumen microbial environment, gastrointestinal tract, general metabolism, and udder. Furthermore, some components are inhaled by the process of eructation [3].

Volatile molecules can derive from several sources, as [3]:

- Carbohydrates.
- Aminoacids.
- Fatty acids.
- Terpenes.
- Other molecules

Although, several classes of compounds have been detected in raw milk [3,4,5,6].

During the physiological modification timelapse, a number of class of molecules have been identified, such as alcohols, ketones, esters, acids, solforates, and hydrocarbons. These molecules present a common presence in the milk samples and species-specific compounds [2]. The volatile profiles (volatilomes) in Grana Padano and Parmigiano-Reggiano have been well defined in these seasoned cheeses, in order to characterize them, defining a volatile fingerprint [7]. These cheeses, broadly diffused in Italy and all over the world, are, not withstanding their apparent similarities, different from the chemical and organoleptic points of view. These differences are dictated by the production protocols, but the raw milk used in cheese production could itself have different physicochemical properties, permitting the evolution of transformations over the ripening cheese process.

Aiming to evaluate more precisely the milks for the production of Grana Padano and Parmigiano-Reggiano from the volatile point of view, a study taking into account the milk destination and the season (spring-summer) was performed by analyzing the volatile content of milk, determined by headspace solid phase micro-extraction gas chromatography mass spectrometry.

## 2. Materials and Methods

A total number of 25 Holstein lactating cows was enrolled: In total, 15 subjects came from three farms for the production of Grana Padano cheese (Cremona, Italy), 10 from two farms producing milk for the caseification of Parmigiano Reggiano (Piacenza, Italy). All cows were multiparous (3–4 years, 5–8 weeks in milk) and submitted to regular veterinary examinations. At the moment of the visit, the cows were considered healthy. Cows were submitted to diets adherent to the disciplinary prescribed to each cheese production. The summary data for each diet are shown in Table 1.

Milk samples were collected every month of the year 2017 in the first teen days of the spring-summer months (May-September), during the morning milking. Samples were refrigerated (4–5 °C) soon after collection and carried to the laboratory for the analysis.

A panel of 54 volatile molecules was determined by headspace analysis as described by Panseri et al. [8]; the molecules analyzed were:

1. 2-Methylbutanal.
2. 2-Propanone.
3. 2-Butanone.
4. 2,3-Butanedione.
5. 2,3-Heptanedione.
6. 2-Heptanone.
7. 3-Hydroxy-2-butanone.
8. 2-Hydroxy-3-pentanone.
9. 2-Nonanone.
10. 5-Hydroxydimehyl-4-octanone.
11. Methanol.
12. 2-Propanol.
13. Ethanol.
14. 2-Methyl-1-propanol.
15. 4-Methyl-2-pentanol.
16. 1-Butanol.
17. 2-Hexanol.
18. 3-Methyl-1-butanol.
19. 3-Methylbut-3-ene-1-ol (isoprenol).
20. 1-Pentanol.
21. 3-Methyl-2-butenol.
22. 3-Pentanol.
23. 1-Hexanol.
24. 2-Hydroxy-3-pentanone 2.
25. 1-Octen-3-ol.
26. 1-Octen-4-ol.
27. 2-Heptanol.
28. 2,3-Butanediol.
29. 2-Butanol.
30. Benzene ethanol.
31. 4-Methyl-2-oxovaleric acid.
32. Ethanoic acid.
33. 2-Methylpropanoic acid.
34. Butanoic acid.
35. Pentanoic acid.
36. Hexanoic acid.
37. Acetic acid methylester.
38. Ethylacetate.
39. Butyric acid methyl ester.
40. Methyl-3-metilbutanoate.
41. Acetic acid ethylenester.
42. Isoamylacetate.
43. Hexanoic acid ethyl ester.
44. Hexanoic acid methyl ester.
45. Isoamyl-n-butyrate.
46. Octanoic acid methyl ester.
47. Methyldecanoate.
48. Butyric acid-3-methyl ester.
49. Hexanoic acid methylester.
50. o-Xylene.
51. p-Xylene.
52. Dimethylsulfide.
53. Dimethylsulfoxide.
54. Dimethylsulfone.

All molecules were subdivided into seven classes, defined following the chemical classes of:

1. Aldehydes.
2. Ketones.
3. Alcohols.
4. Acids.
5. Esters.
6. Aromatic hydrocarbons.
7. Solforates.

The volatile compounds of milk were analyzed according to Panseri et al. [8]. The technique applied was as follows:

### 2.1. HS-SPME Extraction

All milk samples were prepared by weighing exactly 10 mL of milk sample in a 20 mL glass vial, fitted with a cap equipped with silicon/polytetrafluoretylene (PTFE) septa (Supelco, Bellefonte, PA, USA), and by adding 1 mL of the internal standard solution (IS) in water (4-methyl-2-pentanone 1 µg/mL). At the end of the sample equilibration period (1 nh), a conditioned (1.5 h at 280 °C) 1 µL carboxentm/polydimethylsiloxane (CAR/PDMS) StableFlextm fiber (Supelco; Bellefonte, PA, USA) was exposed to the headspace of the sample for extraction (3 h) by a CombiPAL system injector autosampler (CTC analytics, Zwingen, Switzerland). A temperature of 4 °C was selected as the extraction temperature in order to prevent possible matrix alterations (oxidation of some compounds, particularly aldehydes). To keep a constant temperature during analysis, the vials were maintained on a heater plate (CTC Analytics, Zwingen, Switzerland).

### 2.2. Gas Chromatography-Mass Spectrometry Analysis of Volatile Organic Compounds (VOCs)

HS-SPME analysis was performed using a Trace GC Ultra (Thermo-Fisher Scientific; Waltham, MA, USA) Gas Chromatograph coupled to a quadrupole Mass Spectrometer Trace DSQ (Thermo-Fisher Scientific; Waltham, MA, USA) and equipped with an Rtx-Wax column (30 m; 0.25 mm i.d.; 0.25 µm film thickness, Restek, USA). The oven temperature program was: From 35 °C, hold 8 min, to 60 °C at 4 °C/min, then from 60 °C to 160 °C at a rate of 6 °C/min, and finally from 160 °C to 200 °C at a rate of 20 °C/min. Carry over and peaks originating from the fiber were regularly assessed by running blank samples. After each analysis, fibers were immediately thermally desorbed in the GC injector for 5 min at 250 °C to prevent contamination. The injections were performed in splitless mode (5 min). The carrier gas was helium at a constant flow of 1 mL min^-1^. The transfer line to the mass spectrometer was maintained at 230 °C, and the ion source temperature was set at 250 °C. The mass spectra were obtained by using a mass selective detector with the electronic impact at 70 eV, a multiplier voltage of 1456 V, and by collecting the data at rate of 1 scan s^−1^ over the m/z range of 30–350. Compounds were identified by comparing the retention times of the chromatographic peaks with those of authentic compounds analyzed under the same conditions when available, or by comparing the Kovats retention indices with the literature data. The identification of MS fragmentation patterns was performed either by comparison with those of pure compounds or using the National Institute of Standards and Technology (NIST) MS spectral database. The quantitative evaluation was performed using the internal standard procedure, assuming a response factor of one. Thus, the quantitative results (µg/g) of each volatile compound were computed by relating the peak intensity of the volatile compounds to the intensity of the internal standard added to the sample in a known amount.

Data are shown as mean ± standard deviation. The effects of the cheese destination and month of collection were analyzed by the two-way analysis of variance (ANOVA), considering destination and season as fixed factors. Due to the nature of the data, ANOVA was performed on natural logarithmic data, calculated as:

***y’* = *ln* (*y* + 1),**

where *y’* is the new value of the variable, *y* is the original value. The term, *y* + 1, was introduced in order to avoid errors in the logarithm calculation due to zero values.

In order to evaluate possible multivariate differences between the destination and month groups, a multiple factor analysis (MFA), considered as an evolution of principal component analysis, was applied. In this way, multivariate relations between two groups of qualitative variables (month and milk destination) and a group of response variables (volatile classes) were calculated.

In order to better refine the characteristics of the volatile composition of milks, the MFA procedure was extended—for Padano and Reggiano groups—to all volatiles, putting in evidence the correlations of all variables with the first two dimensions, and the correlations of groups with the abovementioned dimensions. Single volatile milk contents were processed by a two-way ANOVA on log-values as described. Month and milk destination were set as fixed factors, and the statistical significance was set at *p* < 0.05.

The univariate descriptive statistics and the ANOVA were performed with the software, JMP ver. 7.0, while the MFA analysis with the software, R ver. 3.5.2. for Windows platform.

## 3. Results

The mean values, the standard deviations, and the effects calculated by ANOVA are reported in Table 2.

ANOVA results underline that the period, at least in the spring-summer period, has a deep influence on the volatile levels, since each volatile class differs significantly during that time. In particular, aldehydes, acids, and aromatic hydrocarbons increased in concentration in late spring-early summer, while ketones, alcohols, esters, and solforate molecules reached their zenith in August (Table 2). When milk destination was considered as a factor, statistical evidence is seen for aldehydes, alcohols, and acids, more abundant in the Parmigiano-Reggiano destined milk, while aromatic hydrocarbons are more present in the Grana Padano destined milk (Table 2). Although not statistically significant, a slight difference in the solforates mean values is evident for Grana Padano milk (Table 2).

**Figure 1:**
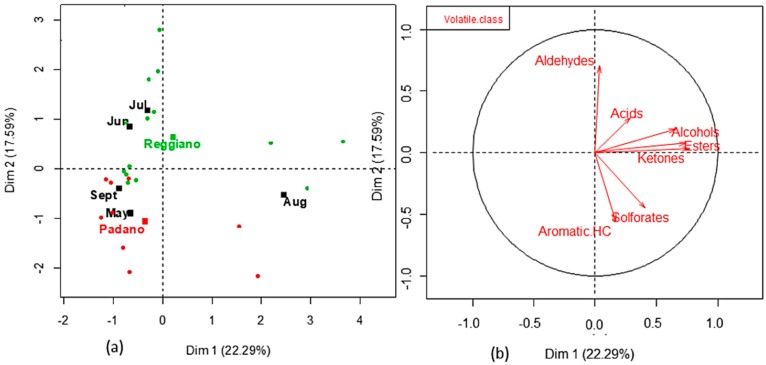
Projection of factors (month-milk destination) **(a)**, and the volatile class **(b)** in the first two dimensions calculated with the MFA technique. Aromatic HC: aromatic hydrocarbons.

The MFA analysis put in evidence the interesting multivariate relation between volatile classes and factors; the first two dimensions explain 39.88% of the total variability (Figure 1). Grana Padano and Parmigiano-Reggiano milk samples (Figure 1a) separate themselves well in the multivariate space, as well as the months of milk collection. In particular, the June-July months form a doublet in the multivariate plane, and the spring-summer months (May-June) form another group of months. An evident singleton is represented by August (Figure 1a). Figure 1b highlights the relation between the quantitative variables (volatile classes): Alcohols, esters, and ketones correlate positively in the first two component planes, while aldehydes and aromatic hydrocarbons, negatively related each other, appear uncorrelated with respect to the abovementioned volatiles. Acids and solforates lay in an intermediate field. From the paired examination of Figure 1a,b, it seems that “mild” months, intended as climatically favorable, are characterized by milks slightly richer in aromatic hydrocarbons and solforates, while August milk, as reported also in the univariate statistics, is abundant in solforates and compounds, such as alcohols, ketones, and acids, and poor in aldehydes.

The univariate distinction between the two different destination milks is repeated also with MFA: Grana Padano samples tend to be richer in solforates and aromatic hydrocarbons with respect to Parmigiano-Reggiano milk, characterized mainly by an abundance of aldehydes. The two milk groups are quite well separated by the zero abscissa of the two-dimensional component plan.

The MFA data processing put in evidence again the sharp separation between the milk groups. Table 3 reports the results for the first two dimensions calculated by MFA, the only dimensions in which the separation between the milks is statistically significant.

By examining dimension 1, Parmigiano milk correlates well with the presence of several esters, alcohols, and ketones, whereas Padano milk appears inversely related with these components (Table 3). The negative correlation between Padano milk and dimension 1 evidences positive correlations with this type of milk and the presence of p-xylene (negatively correlated with dimension 1) (Table 3).

Dimension 2 relates positively with Padano milk, except for methanol (Table 3). Also, in this case, several branched esters and alcohols can be appreciated and, notably, dimethylsulfide, directly related to Padano milk group.

The effects of the month of production and destination on the single volatile levels are reported in Table 4. It is clear that the month of production can heavily influence the volatile levels, while the types of milk differ only in four molecules (2-methylbutanal, 2,3-butanedione, 2-propanol, p-xylene). So, multivariate analysis seems to uncover relations between molecules in a more complete way than univariate statistics.

## 4. Discussion

This survey on the volatile characteristics of milk destined for the production of two broadly diffused cheeses evidences differences between products before transformation. From our results, the year period can heavily influence the abundance or lack of substances, according to [9]. The presence of certain classes of volatiles is the result of a complex interaction between the physiological status of forages/grazing, the environment, and the metabolism of the cow. Therefore, it might be very difficult to separate these sources to allow forecasting of, e.g., the characteristics of a given subject metabolism or the influence of a given pasture, but the selection of a group of volatile molecules in milk could characterize several aspects of ruminant physiology. The differences in the two groups of milk samples destined for the production of Grana Padano and Parmigiano-Reggiano could primarily be due to the different production methods (e.g., forbidden use of silages in Parmigiano-Reggiano production, used instead in Grana Padano), although the significant differences in volatiles could be due to the broad dispersion of data, as evidenced by the ample standard deviations, and ANOVA analysis was performed on log-transformed data. The month of milking seems then to influence in a more marked fashion the volatile composition with respect to the milk destination groups, maybe due to variations in hay and fresh forage seasonality or metabolic reactions to higher temperatures. The multivariate analysis (MFA) on single volatile molecules, on the opposite side, showed a quite different pattern, leading to evidence of a net distinction between the two types of milk seen in a multidimensional space. Some milk components (more or less volatile) can have a great impact on the transformed product following ripening and the action of bacterial microflora, even if these modifications in cheese or milk are often missed by untrained consumers that cannot easily perceive these differences [10,11].

Parmigiano-Reggiano milk contains higher concentrations of aldehydes, alcohols, and acids than milk destined for Grana Padano production. Aldehydes as a pentanal can contribute to the appearance of grassy aromas, mainly when feed derives from pastures with respect to concentrates [12]. An inverse trend is generally reported for alcohols, ethanol in particular, which are mainly concentrated in silage-fed animals. Our results seem to follow an opposite direction, since Parmigiano-Reggiano milk was found to be richer in alcohols, but was milked from cows in which silage feeding of cows is forbidden. This feature could suggest a non-alimentary source of alcohols. Organic acids could derive, at least in part, directly from feed. It is reported [13] that higher levels of carboxylic acids are found in animals fed with hay with respect to pasture, probably because of a concentration effect. However, [14] presents a review with contrasting results about this aspect.

The picture of volatile compounds for milk before cheesemaking could add precious information about the physiological condition of animals, as well as help to make forecasts on the evolution of transformed products, as a predictor of cheese quality and characterization.

## Figures and Tables

**Table 1:** Parameters for the dietary treatments for the two groups of cows.

Parameter	Grana Padano (%)	Parmigiano Reggiano (%)
Crude protein	15.36	15.94
Ethereal extracts	4.10	3.44
Ash	7.05	8.60
Nitrogen disappearance rate	54.75	66.68
Fiber	16.80	4.62
Ca	0.75	0.9
P	0.56	1.02
Na	0.50	1.75
	Alfalfa hay	Alfalfa hay
	Soy meal	Polyphyte hay
	Maize silage	Maize grain
	Sunflower meal	Soy meal
	Corn flakes	Sunflower meal
	Barley flakes	Wheat bran
	Wheat bran	Soybean hulls
	Molasses	Molasses
	Minerals	Minerals
	Hydrogenated fat	

**Table 2:** Volatiles concentrations (mean ± standard deviation, ng/gEq) for the seven classes of chemicals, subdivided for month and milk destination. The table reports the *p*-values for ANOVA. SD: standard deviation.

Volatile	Month					ANOVA Month	Destination		ANOVA Destination
	May	Jun	Jul	Aug	Sept		Padano	Reggiano	
Aldehydes	36.56	433.76	418.96	79.89	48.16	<0.05	24.09	169.55	<0.05
SD	34.57	664.44	264.89	73.52	27.65		45.23	310.76	
Ketones	459.62	644.99	791.98	1774.09	276.54	<0.001	441.55	658.01	-
SD	255.00	557.30	371.18	1571.43	184.59		749.95	828.72	
Alcohols	325.15	1385.07	1843.71	12,133.02	211.11	<0.001	308.04	2649.70	<0.05
SD	342.34	1629.67	1010.86	7893.76	71.54		439.30	5223.71	
Acids	91.72	979.42	426.48	342.64	42.63	<0.001	205.64	313.82	<0.05
SD	126.65	1329.30	243.26	205.97	24.63		624.56	584.32	
Esters	55.20	129.78	91.33	10,006.10	3.44	<0.001	129.88	1714.44	-
SD	10.89	207.39	65.74	9291.23	1.00		166.50	4972.23	
Aromatic hydrocarbons	0.00	0.11	0.00	0.00	0.00	<0.01	1.26	0.02	<0.01
SD	0.00	0.20	0.00	0.00	0.00		2.59	0.08	
Solforates	16.57	1.13	8.99	40.33	29.50	<0.01	21.94	16.09	-
SD	2.51	1.01	12.28	69.83	6.83		27.17	28.76	

**Table 3:** Correlation summary for the first five dimensions of MFA analysis performed on single volatiles. Only significant correlations for each dimension are reported. The table also reports the correlations of the two destination milk groups (Parmigiano Reggiano or Grana Padano) with the dimensions.

Dimension 1 (27.13%)			Dimension 2 (17.06%)			Dimension 3 (10.79%)		
	Correlation	*p*-Value		Correlation	*p*-Value		Correlation	*p*-Value
3-methyl-1-butanol	0.64	0.001	3-methyl-2-butenol	0.78	<0.0001	1-octen-3-ol	0.9356	<0.0001
2,3-eptanedione	0.58	0.003	2,3-butanediol	0.77	<0.0001	2-hydroxy-3-pentanone	0.9353	<0.0001
3-methylbut-3-ene-1-ol	0.5	0.003	ethylacetate	0.73	<0.0001	acetic acid methylester	0.9106	<0.0001
ethylacetate	0.57	0.003	3-hydroxy-2-butanone	0.73	0.0001	ethanol	0.9083	<0.0001
3-methyl-2-butenol	0.57	0.003	butyric acid methylester	0.69	0.0002	pentanoic acid	0.8991	<0.0001
butyric acid methylester	0.57	0.003	3-methylbut-3-ene-1-ol	0.68	0.0003	benzenethanol	0.7657	<0.0001
2,3-butanedione	0.56	0.003	hexanoic acid methylester	0.66	0.0004	2-methyl-propanol	0.752	<0.0001
hexanoic-acid-ethyl-ester	0.54	0.006	methyldecanoate	0.65	0.0006	hexanoic acid methylester	0.5628	0.0042
1-hexanol	0.53	0.007	hexanoic acid ethylester	0.64	0.0008	3-methyl-1-butanol	0.537	0.0068
isoamylacetate	0.53	0.007	isoamylacetate	0.63	0.001			
2-methyl-1-propanol	0.52	0.007	acetic acid ethylenester	0.61	0.0015			
methyldecanoate	0.51	0.01	methyl 3 methylbutanoate	0.61	0.0016			
acetic acid ethylenester	0.50	0.01	hexanoic acid methyl ester	0.60	0.0021			
octanoic acid methylester	0.50	0.01	2,3- heptanedione	0.5772	0.0031			
3-pentanol	0.49	0.01	3-pentanol	0.5597	0.0045			
2-hydroxy-3-pentanone	0.48	0.01	2-hydroxy-3-pentanone	0.5352	0.007			
hexanoic acid methylester	0.47	0.02	octanoic acid methylester	0.4815	0.0172			
3-hydroxy-2-butanone	0.44	0.03	dimethylsulfide	0.4244	0.0387			
hexanoic acid methylester	0.43	0.033	methanol	–0.4503	0.0272			
1-octen-4-ol	0.4318	0.03						
p-xylene	−0.4199	0.04						
**Destination**	**R2**	***p*-value**	**Destination**	**R2**	***p*-value**	**Destination**	**R2**	***p*-value**
	0.77	<0.0001		0.1845	0.0362		n.s.	n.s.
Reggiano	10.5	<0.0001	Padano	0.4079	0.0362	Padano	n.s.	n.s.
Padano	–10.5	<0.0001	Reggiano	–0.4079	0.0362	Reggiano	n.s.	n.s.
**Dimension 4 (9.03%)**			**Dimension 5 (5.86%)**					
	**Correlation**	***p*-value**		**Correlation**	***p*-value**			
2-eptanone	0.76	<0.0001	acetic acid	0.8772	<0.0001			
2-methylpropanoic acid	0.75	<0.0001	o-xylene	0.847	<0.0001			
dimethylsulfide	0.71	0.0001	1-butanol	0.8295	<0.0001			
butyric acid-3-methylester	0.53	0.0076	methyl-3-methylbutanoate	0.5721	0.0035			
octanoic acid methylester	0.51	0.0094						
1-octen-4-ol	0.50	0.0121						
2-hydroxy-3-pentanone	0.4	0.0133						
3-methyl-1-butanol	0.4054	0.0493						
hexanoic acid ethylester	–0.43	0.0334						
2-3-eptanedione	–0.45	0.0251						
isoamylacetate	–0.49	0.0143						
methyldecanoate	–0.50	0.0119						
1-hexanol	–0.54	0.0057						
hexanoic acid methylester	–0.54	0.0056						
acetic acid etilenester	–0.55	0.0053						
**Destination**	**R2**	***p*-value**	**Destination**	**R2**	***p*-value**			
	n.s.	n.s.		n.s.	n.s.			
Padano	n.s.	n.s.	Padano	n.s.	n.s.			
Reggiano	n.s.	n.s.	Reggiano	n.s.	n.s.			

**Table 4:** Descriptive statistics and two-way ANOVA results for the volatile molecules in Reggiano and Padano destined milks. The asterisks indicate significance with *p* between 0.05 and 0.1.

	Padano						Reggiano					Mean Volatiles			
Volatile	Statistics	May	Jun	Jul	Aug	Sept	May	Jun	Jul	Aug	Sept	ANOVA Month	Padano	Reggiano	ANOVA Milk
2-methylbutanal	Mean	8.53	3.12	12.83	48.81	65.63	36.56	433.76	418.96	79.89	48.16	-	24.09	16.,55	<0.05
	Std Dev	0.00	4.42		69.03	91.35	34.57	664.44	264.89	73.52	27.65		45.23	310.76	
2-propanone	Mean	30.43	10.94	20.88	12.36	28.16	222.51	21.88	77.05	56.22	52.56	<0.01	16.82	71.74	-
	Std Dev	6.85	0.90		17.48	39.83	280.64	19.02	59.43	97.38	19.70		18.02	127.86	
2-butanone	Mean	10.87	11.85	36.95	0.00	0.00	9.08	0.00	3.45	0.00	17.82	-	7.54	5.06	-
	Std Dev	12.22	0.85		0.00	0.00	11.38	0.00	3.00	0.00	15.90		11.86	9.57	
2,3-butanedione	Mean	12.85	0.40	10.44	4.90	10.56	2.82	46.32	27.36	37.57	13.47	<0.05	6.17	21.26	<0.05
	Std Dev	13.23	0.56		6.93	12.63	2.67	32.39	12.54	24.88	14.05		8.21	23.53	
2,3-heptanedione	Mean	0.00	0.00	0.00	1.44	0.00	7.54	6.91	4.85	31.16	0.00	-	0.26	8.41	*
	Std Dev	0.00	0.00		2.04	0.00	13.07	10.76	4.31	47.39	0.00		0.87	20.47	
2-heptanone	Mean	1.72	0.64	0.52	2.71	0.48	0.79	1.43	3.00	13.27	1.12	-	1.06	3.27	-
	Std Dev	1.36	0.05		2.45	0.38	0.90	1.26	1.51	22.98	0.90		1.34	9.21	
3-hydroxy-2-butanone	Mean	270.47	37.91	206.83	1671.07	46.19	216.65	557.59	670.74	1632.59	187.73	<0.001	387.11	544.31	-
	Std Dev	169.11	45.94		1211.05	56.70	185.95	541.34	373.89	1507.99	165.44		750.55	794.46	
2-hydroxy-3-pentanone	Mean	1.70	0.32	1.80	4.60	0.23	0.00	3.71	3.80	8.45	0.00	<0.05	1.41	2.66	-
	Std Dev	1.18	0.45		2.27	0.32	0.00	2.71	4.01	8.42	0.00		1.91	4.60	
2-nonanone	Mean	8.74	0.33	0.00	0.00	0.00	0.22	0.00	1.72	0.00	3.84	-	1.65	0.96	-
	Std Dev	0.14	0.47		0.00	0.00	0.39	0.00	2.98	0.00	6.66		3.51	2.90	
5-idrossidimetil-4-ottanone	Mean	107.41	0.00	0.00	0.00	0.00	0.00	7.14	0.00	0.00	0.00	-	19.53	1,19	-
	Std Dev	142.99	0.00		0.00	0.00	0.00	12.37	0.00	0.00	0.00		62.71	5,05	
Methanol	Mean	0.00	0.00	0.00	0.00	0.00	0.00	38.06	11.32	0.00	18.53	<0.05	0.00	11.32	*
	Std Dev	0.00	0.00		0.00	0.00	0.00	9.86	10.33	0.00	24.37		0.00	17.23	
2-propanol	Mean	0.00	0.00	0.00	0.00	0.00	5.13	19.93	54.48	0.00	1.47	-	0.00	13.50	<0.01
	Std Dev	0.00	0.00		0.00	0.00	8.88	34.52	94.36	0.00	2.54		0.00	40.04	
Ethanol	Mean	170.06	18.85	39.42	377.55	0.00	18.93	37.99	31.89	5175.42	44.00	<0.001	106.58	884.71	-
	Std Dev	166.68	17.29		305.12	0.00	32.79	54.80	30.21	5687.78	42.97		184.78	2775.90	
2-methyl-1-propanol	Mean	11.91	0.49	1.79	26.87	0.22	21.03	19.53	42.34	250.06	0.88	<0.001	7.35	55.64	-
	Std Dev	5.33	0.33		4.13	0.31	14.47	32.31	27.74	161.83	0.88		10.88	107.42	
4-methyl-2-pentanol	Mean	0.33	0.02	0.00	5.02	0.00	0.00	0.00	0.00	0.00	0.00	-	0..8	0.00	-
	Std Dev	0.46	0.02		7.09	0.00	0.00	0.00	0.00	0.00	0.00		3..1	0.00	
1-butanol	Mean	0.00	0.00	0.00	0.56	0.00	0.00	0.00	0.00	0.00	0.14	-	0..0	0.02	-
	Std Dev	0.00	0.00		0.80	0.00	0.00	0.00	0.00	0.00	0.25		0.34	0.10	
2-hexanol	Mean	0.63	0.00	0.00	0.00	0.00	0.18	0.00	0.00	0.00	0.00	<0.5	0.12	0.03	-
	Std Dev	0.60	0.00		0.00	0.00	0.31	0.00	0.00	0.00	0.00		0.31	0.13	
3-methyl-1-butanol	Mean	190.67	59.84	107.83	568.10	25.31	269.91	884.81	1451.82	6065.24	86.11	<0.001	163.25	1459.66	°
	Std Dev	167.41	82.53		217.28	5.07	301.79	1037.11	918.35	2069.45	35.84		229.60	2345.35	
3-methylbut-3-ene-1-ol	Mean	0.33	0.06	0.31	0.95	0.33	1.47	0.00	0.11	4.76	0.58	<0.05	0.33	1.16	*
	Std Dev	0.04	0.08		1.34	0.03	0.23	0.00	0.20	4.13	0.55		0.54	2.26	
1-pentanol	Mean	0.30	0.06	0.31	0.00	0.55	0.00	0.19	0.45	0.00	0.60	<0.05	0.19	0.21	-
	Std Dev	0.18	0.08		0.00	0.33	0.00	0.33	0.78	0.00	0.26		0.25	0.39	
3-methyl-2-butenol	Mean	0.00	0.00	0.25	1.81	0.06	0.00	0.49	0.29	5.62	0.27	<0.01	0.36	1.11	-
	Std Dev	0.00	0.00		0.05	0.08	0.00	0.42	0.50	5.37	0.24		0.72	2.79	
3-pentanol	Mean	1.62	0.07	3.51	6.41	0.60	1.35	4.76	4.08	10.47	0.20	<0.001	1.90	3.48	-
	Std Dev	1.35	0.10		3.17	0.65	1.13	7.42	2.33	6.92	0.35		2.70	5.17	
1-hexanol	Mean	0.00	0.13	0.35	0.00	0.22	0.90	1.21	1.91	1.63	0.71	-	0.10	1.06	-
	Std Dev	0.00	0.10		0.00	0.02	1.56	1.62	1.73	2.82	0.62		0.13	1.53	
2-hydroxy-3-pentanone 2	Mean	0.00	0.00	0.00	0.00	0.00	0.74	0.00	0.00	10.36	0.00	-	0.00	1.85	-
	Std Dev	0.00	0.00		0.00	0.00	1.28	0.00	0.00	17.94	0.00		0.00	7.31	
1-octen-3-ol	Mean	0.00	0.06	5.84	0.00	0.00	2.27	0.00	0.00	257.85	0.00	-	0.54	43.35	-
	Std Dev	0.00	0.01		0.00	0.00	3.93	0.00	0.00	446.61	0.00		1.76	182.24	
1-octen-4-ol	Mean	0.00	1.21	1.14	41.68	0.24	0.00	331.66	227.37	241.98	0.38	<0.01	7.95	133.56	-
	Std Dev	0.00	1.58		55.38	0.34	0.00	525.86	207.31	343.34	0.66		24.20	267.27	
2-heptanol	Mean	0.00	0.29	0.00	0.00	4.87	0.23	6.86	0.00	2.54	1.28	-	0.94	1.82	-
	Std Dev	0.00	0.29		0.00	6.89	0.40	11.88	0.00	4.40	2.22		2.92	5.07	
2,3-butanediol	Mean	0.00	0.00	0.00	14.74	0.09	0.00	0.00	0.00	9.75	0.49	<0.01	2.70	1.71	-
	Std Dev	0.00	0.00		7.71	0.13	0.00	0.00	0.00	16.88	0.85		6.43	6.88	
2-butanol	Mean	0.10	0.00	0.00	53.21	0.00	0.00	2.87	2.86	3.32	0.00	-	9.69	1.51	-
	Std Dev	0.14	0.00		75.25	0.00	0.00	3.26	4.95	5.75	0.00		32.08	3.23	
Benzene ehtanol	Mean	0.50	0.77	1.39	25.37	0.00	3.01	36.72	14.80	94.01	55.48	-	4.97	34.00	-
	Std Dev	0.71	1.09		35.88	0.00	3.86	34.55	15.31	162.81	83.03		15.19	72.50	
4-methyl-2-oxovaleric acid	Mean	0.71	0.37	1.90	18.83	0.24	3.14	104.72	55.93	69.18	0.47	<0.001	3.84	38.91	-
	Std Dev	0.71	0.52		9.57	0.34	5.44	163.45	47.20	55.42	0.41		8.03	74.17	
Ethanoic acid	Mean	1.77	0.00	0.00	929.79	0.20	58.89	23.64	189.16	28.77	0.00	-	169.41	50.08	-
	Std Dev	0.68	0.00		1314.92	0.28	79.68	34.57	167.19	49.83	0.00		560.56	94.78	
2-methylpropanoic acid	Mean	0.00	0.00	9.36	0.00	0.00	2.56	5.82	0.00	84.84	0.51	-	0.85	15.62	-
	Std Dev	0.00	0.00		0.00	0.00	4.43	10.08	0.00	146.95	0.45		2.82	59.78	
Butanoic acid	Mean	16.18	7.10	0.00	74.35	7.24	4.22	827.63	23.03	52.46	18.60	-	19.07	154.33	-
	Std Dev	22.88	5.53		105.14	0.42	4.09	1153.83	27.35	90.87	19.82		44.04	504.02	
Pentanoic acid	Mean	0.00	0.00	0.00	7.82	38.36	22.91	17.60	16.62	89.90	3.74	-	8.40	25.13	-
	Std Dev	0.00	0.00		11.06	50.77	34.36	30.49	28.79	155.71	0.72		22.34	64.45	
Hexanoic acid	Mean	0.00	0.00	0.00	31.67	3.52	0.00	0.00	141.74	17.49	19.31	<0.05	6.40	29.76	-
	Std Dev	0.00	0.00		44.79	2.26	0.00	0.00	160.94	30.29	21.35		18.95	77.05	
acetic acid methylester	Mean	18.20	8.53	0.00	21.35	25.08	36.80	5.93	11.59	829.83	0.00	<0.01	13.36	147.42	-
	Std Dev	8.55	12.06		16.10	29.52	20.97	10.27	13.87	1014.77	0.00		15.37	469.05	
Ethylacetate	Mean	196.72	22.03	0.00	45.53	0.00	9.13	6.76	52.51	6654.61	0.00	<0.05	48.06	1120.56	-
	Std Dev	111.47	31.16		64.39	0.00	15.81	11.71	37.52	7413.99	0.00		86.40	3598.99	
Butyric acid methyl ester	Mean	0.00	0.91	0.00	5.61	0.43	0.00	0.39	2.41	53.09	0.00	<0.05	1.27	9.32	-
	Std Dev	0.00	1.29		7.93	0.61	0.00	0.68	4.17	46.36	0.00		3.35	25.72	
Methyl-3-metilbutanoate	Mean	0.00	0.00	0.00	63.44	28.82	4.49	0.00	0.00	57.67	0.00	<0.01	16.77	10.36	-
	Std Dev	0.00	0.00		89.72	40.75	7.77	0.00	0.00	25.77	0.00		40.40	23.71	
acetic acid ethylenester	Mean	0.01	0.00	0.00	2.43	0.00	2.13	0.00	6.48	26.42	1.14	-	0.44	6.03	-
	Std Dev	0.01	0.00		3.44	0.00	3.70	0.00	11.22	45.75	1.98		1.46	18.87	
Isoamylacetate	Mean	7.28	0.26	0.28	26.88	0.12	0.99	0.00	13.54	1853.35	0.00	<0.01	6.31	311.31	-
	Std Dev	4.45	0.37		38.02	0.16	0.96	0.00	16.37	2378.68	0.00		16.06	1081.33	
Hexanoic acid ethyl ester	Mean	0.73	1.00	0.68	4.59	0.72	1.66	4.59	4.82	14.48	0.50	<0.05	1.35	4.34	-
	Std Dev	0.18	0.27		0.80	0.13	1.07	6.50	2.96	19.84	0.48		1.66	8.82	
Hexanoic acid methyl ester	Mean	0.50	0.00	0.00	30.71	1.07	0.00	0.00	0.00	61.78	1.52	<0.001	5.87	10.55	-
	Std Dev	0.71	0.00		36.17	0.83	0.00	0.00	0.00	43.59	0.48		16.79	27.92	
isoamyl-n-butyrate	Mean	0.06	0.00	0.00	0.00	0.00	0.00	0.00	0.00	0.00	0.00	-	0.01	0.00	-
	Std Dev	0.09	0.00		0.00	0.00	0.00	0.00	0.00	0.00	0.00		0.04	0.00	
Octanoic acid methyl ester	Mean	0.00	0.22	0.57	0.00	0.00	0.00	2.16	0.00	18.31	0.28	-	0.09	3.46	-
	Std Dev	0.00	0.31		0.00	0.00	0.00	3.27	0.00	20.67	0.49		0.21	9.94	
Methyldecanoate	Mean	1.16	0.09	0.00	12.88	0.00	0.00	0.00	0.00	180.01	0.00	<0.01	2.57	30,00	-
	Std Dev	1.64	0.01		18.22	0.00	0.00	0.00	0.00	243.36	0.00		7.72	108.32	
Butyric acid-3-methyl ester	Mean	0.00	0.00	0.00	172.33	0.00	0.00	109.94	0.00	145.61	0.00	<0.05	31.33	42.59	-
	Std Dev	0.00	0.00		243.71	0.00	0.00	190.43	0.00	211.31	0.00		103.92	116.07	
Hexanoic acid methylester	Mean	0.72	2.11	0.00	0.00	0.00	0.00	0.00	0.00	58.55	0.00	-	0.52	9.76	-
	Std Dev	1.02	2.98		0.00	0.00	0.00	0.00	0.00	101.42	0.00		1.30	41.40	
o-xylene	Mean	0.13	0.00	0.00	2.58	0.00	0.00	0.11	0.00	0.00	0.00	-	0.49	0.02	-
	Std Dev	0.18	0.00		3.65	0.00	0.00	0.20	0.00	0.00	0.00		1.55	0.08	
p-xylene	Mean	3.04	0.00	0.00	1.17	0.00	0.00	0.00	0.00	0.00	0.00	-	0.77	0.00	<0.001
	Std Dev	3.06	0.00		1.66	0.00	0.00	0.00	0.00	0.00	0.00		1.64	0.00	
Dimethylsulfide	Mean	21.73	7.30	35.46	56.01	1.80	14.53	0.00	7.67	40.32	9.69	<0.05	19.01	12.03	-
	Std Dev	5.32	6.54		42.67	2.55	4.25	0.00	13.27	69.83	13.29		25.51	28.55	
Dimethylsulfoxide	Mean	0.00	0.21	0.00	2.49	0.00	0.00	1.13	0.00	0.00	6.32	-	0.49	1.24	
	Std Dev	0.00	0.30		3.52	0.00	0.00	1.01	0.00	0.00	8.21		1.49	3.70	
Dimethylsulfone	Mean	0.81	2.11	0.87	2.23	7.80	2.04	0.00	1.32	0.01	13.49	<0.001	2.44	2.81	-
	Std Dev	0.26	1.00		3.16	5.96	1.83	0.00	2.29	0.01	5.63	-	3.52	5.43	-
